# Entropy-adaptive differential privacy federated learning for student performance prediction and privacy protection: a case study in Python programming

**DOI:** 10.3389/frai.2025.1653437

**Published:** 2025-09-08

**Authors:** Shanwei Chen, Xiuzhi Qi

**Affiliations:** ^1^College of Education, Baoji University of Arts and Sciences, Baoji, China; ^2^Academy of Fine Arts, Baoji University of Arts and Sciences, Baoji, China

**Keywords:** federated learning, entropy-adaptive differential privacy, student performance prediction, distributed data analysis, Python programming

## Abstract

In the context of the digital transformation of engineering education, protecting student data privacy has become a key challenge for enabling data-driven instruction. This study proposes an Entropy-Adaptive Differential Privacy Federated Learning method (EADP-FedAvg) to enhance the accuracy of student performance prediction while ensuring data privacy. Based on online test records from Python programming courses for Electronic Engineering students (grade 2021–2023) at the School of Physics and Optoelectronic Technology, Baoji University of Arts and Sciences, China, the study uses a Multilayer Perceptron (MLP) model and 10 distributed clients for training. Under different privacy budgets (*ε* = 0.1, 1e-6, and 1.0), EADP-FedAvg achieves a test accuracy of 92.7%, macro-average score of 92.1%, and entropy of 0.207, outperforming standard federated learning and approaching centralized learning performance. The results demonstrate that by adaptively adjusting the noise level based on output entropy, EADP-FedAvg effectively balances privacy preservation and model accuracy. This method offers a novel solution for analyzing privacy-sensitive educational data in engineering education.

## 1 Introduction

The digital transformation of engineering education has led to an unprecedented increase in student data, offering valuable opportunities for optimizing instructional strategies and enabling personalized learning. These datasets, ranging from learning behaviors and academic performance to interaction records, form the foundation for analyzing student outcomes and improving teaching quality (Shou et al., 2024). In many undergraduate engineering programs, Python Programming is a core course that cultivates computational thinking and coding skills. Its delivery via online platforms generates large-scale structured datasets, making it an ideal setting for data-driven educational research. However, the sensitive nature of student data raises critical privacy concerns, especially under the tightening global data protection regulations and evolving educational ethics standards (Marshall et al., 2022).

To address this challenge, we propose Entropy-Adaptive Differential Privacy Federated Averaging (EADP-FedAvg)—a method designed to improve prediction accuracy in Python programming courses while safeguarding student privacy. Federated Learning (FL) is a decentralized machine learning framework that allows multiple clients to collaboratively train a model without sharing raw data, making it naturally suited for privacy-sensitive educational applications (Moshawrab et al., 2023). However, conventional FL combined with fixed-noise Differential Privacy (DP) often suffers from significant performance degradation due to excessive noise (Elgabli and Mesbah, 2024). EADP-FedAvg tackles this issue by dynamically adjusting noise intensity based on the model’s average output entropy, achieving a more effective balance between privacy and accuracy, even under stringent privacy budgets.

This study uses 2,452 online test records from 493 students majoring in Electronic Engineering (Classes of 2021–2023) at Baoji University of Arts and Sciences. The dataset includes 17 features such as course click counts, assignment scores, and study duration, along with four performance categories: Fail, Passed, Good, and Excellent. The experiments are conducted in a simulated environment using 10 federated clients and a Multilayer Perceptron (MLP) model.

The study focuses on the following research questions:

1. How can FL protect student test data privacy and improve prediction accuracy in Python programming courses?
2. How does entropy-adaptive differential privacy enhance conventional FL by optimizing the privacy-accuracy trade-off?
3. What is the potential of EADP-FedAvg for privacy-preserving data analysis in engineering education?

The remainder of this paper is structured as follows. Section 2 reviews the latest research developments and clarifies the innovative positioning of this study within the fields of FL and privacy preservation. Section 3 presents the data sources and outlines the model architecture. Section 4 describes the experimental setup and provides a detailed explanation of the model design. Section 5 offers a comparative analysis of three models using evaluation metrics such as confusion matrices and information entropy. Finally, Section 6 summarizes the key findings and explores potential directions for future research.

## 2 Related research

### 2.1 FL in engineering education

FL is a decentralized machine learning framework that enables multiple clients to collaboratively train models without sharing their raw data (McMahan et al., 2016). In Python programming courses, students generate a wealth of data through online tests and learning platforms, including performance records, behavioral features, and submission logs. While this data provides valuable resources for predicting student outcomes, traditional centralized machine learning methods require aggregating data on a central server, which raises significant privacy concerns and presents integration challenges due to data fragmentation (Bonawitz et al., 2021). FL addresses these issues by performing localized training and aggregating model parameters, making it particularly suitable for privacy-sensitive educational environments.

In engineering education, FL has already been applied to programming course data analysis. For instance, Christiansen et al. (2023) introduced a federated framework for cross-institutional analysis of programming course data to predict student performance, demonstrating FL’s generalizability in heterogeneous data environments. Similarly, Truex et al. (2019) employed FL to analyze C++ course test data, using distributed training to protect student privacy while maintaining model accuracy. These studies suggest that FL can support data-driven teaching optimization in a privacy-compliant manner.

However, several challenges remain in applying FL to educational data analysis. First, data heterogeneity across clients can hinder model convergence (Mora et al., 2024). Second, when FL is combined with DP, the use of fixed noise levels often degrades prediction performance (Sattler et al., 2019). Third, centralized AI methods that rely on aggregating data into a single location fall short of meeting privacy demands (Song and others, 2023). Lastly, existing FL research has rarely explored adaptive noise mechanisms that dynamically balance privacy protection with model performance.

### 2.2 Privacy-preserving technologies in education

As engineering education undergoes digital transformation, protecting student data privacy has become a cornerstone of data-driven instruction. In many technical courses such as Python Programming, the online test data generated often contains sensitive information which, if mishandled, may lead to privacy breaches. To address this issue, various privacy-preserving technologies have been introduced into educational contexts, including DP, data anonymization, Secure Multi-Party Computation (SMPC), and Homomorphic Encryption (HE). These methods aim to protect student privacy while still supporting high-precision performance prediction, making them particularly well-suited for privacy-sensitive educational environments.

DP ensures that individual data cannot be reverse-engineered by injecting carefully calibrated noise into model outputs or parameters. It is considered the gold standard in educational data analysis (Pakina and Pujari, 2024). In this study, DP can be used to safeguard test scores and behavioral data. For example, (Zhan et al., 2024) proposed a Gaussian mechanism–based DP method for predicting student performance that maintained high accuracy under a privacy budget of *ε* = 0.1. However, traditional DP approaches often apply a fixed noise level, which can significantly degrade model performance—especially when working with small datasets (Afrose et al., 2021).

Data anonymization is a fundamental technique that reduces the risk of individual identification by removing or replacing personally identifiable information (PII), such as student IDs or names. In educational data processing, anonymization allows for meaningful analysis while protecting student identities. For instance, Chicaiza et al. (2020) applied k-anonymity to anonymize learning data from programming courses. This technique generalizes or suppresses sensitive attributes so that each record is indistinguishable from at least k other records based on quasi-identifiers. It enabled cross-class comparison without exposing student identities. However, k-anonymity often leads to information loss—especially in high-dimensional datasets—by reducing the granularity of key features, which can limit deeper data analysis and compromise model performance.

Secure Multi-Party Computation (SMPC) allows multiple parties to jointly compute functions over their data without revealing the data itself. As noted by Khan (2024), this approach is particularly suitable for collaborative research across institutions—such as sharing behavioral data across schools for joint analysis. While SMPC ensures that local data remains private, it requires substantial coordination among parties and may introduce significant communication overhead and system complexity.

HE enables direct computation on encrypted data such that the decrypted result is identical to what would have been obtained using plaintext inputs. The fully homomorphic encryption scheme proposed by Zhang et al. (2024) laid the foundation for this area. In theory, HE allows training and prediction to occur entirely in the encrypted domain, offering the highest level of data privacy. However, current HE methods still face challenges of high computational complexity and low efficiency, making them difficult to deploy in real-time educational scenarios.

### 2.3 Student data privacy in programming courses

The privacy challenges associated with programming course data stem primarily from its high dimensionality and heterogeneity. In courses like Python Programming, online test data often includes numerical features such as scores and discrete features such as click counts. Analyzing such complex data typically requires sophisticated models, which increases the risk of privacy leakage. Traditional methods like data anonymization reduce risk by removing identifiers, but often result in significant information loss, limiting the depth of analysis (Salas and Domingo-Ferrer, 2018). Techniques such as Secure Multi-Party Computation (SMPC) and Homomorphic Encryption (HE) enable privacy-preserving computation but are computationally intensive, making them impractical for real-time analysis of large-scale course data (Liu, 2024).

DP has emerged as the preferred method for protecting student privacy in programming courses by adding noise to safeguard data. For example, Miller and Chattopadhyay (2024) applied DP to test score data in a database course and achieved high prediction accuracy under a privacy budget of *ε* = 0.1. However, the use of fixed-noise DP can degrade model performance, particularly on small datasets. Adaptive DP, which dynamically adjusts the noise level, has demonstrated the ability to strike a better balance between noise and model utility (Yang et al., 2023). In response to these practical challenges, this study proposes the EADP-FedAvg method, which leverages average information entropy to dynamically adjust noise intensity.

## 3 Data and methods

### 3.1 Data definition

The dataset was collected from the Python Programming course offered at Baoji University of Arts and Sciences, School of Physics and Optoelectronic Technology, and includes data from students majoring in Electronic Engineering. Specifically, it covers three academic cohorts: Class of 2021 (four classes), Class of 2022 (four classes), and Class of 2023 (four classes). The course is a mandatory semester-long program aimed at developing students’ programming proficiency and algorithmic thinking. A total of 493 students participated, generating 2,465 test records, of which 2,452 remained after data cleaning.

Each semester includes five online tests, conducted through a Learning Management System (LMS), capturing test scores, learning behavior, and anonymized student demographics. The performance labels are divided into four categories: Fail, Passed, Good, and Excellent.

The total score for each test was 100 points, comprising four types of questions. Multiple-choice questions included 5 items worth 4 points each, totaling 20 points. These primarily assessed students’ understanding of fundamental syntax and core programming concepts, such as variable naming rules, reserved keywords, and operator usage. Fill-in-the-blank questions consisted of 3 items worth 5 points each, totaling 15 points. Students were required to complete specific code snippets, often involving string formatting techniques or loop structures. True/False questions included 5 items worth 3 points each, totaling 15 points. These tested students’ logical reasoning skills, for example, their understanding of increment operations or data type distinctions. Programming problems made up the remaining 50 points across 2 questions. Students were tasked with solving practical coding challenges, such as determining whether a number is even or odd, or implementing a list summation function. Scoring for these items considered the correctness of input handling, logical implementation, and the accuracy of the program output.

Learning behavior features include submission frequency, response time, and error frequency, totaling 10 features that reflect learning patterns and course difficulty.

Student demographic features include gender and age, allowing exploration of background influences on performance.

As shown in Table 1, the dataset contains 17 features: 5 related to scores, 10 to behavioral patterns, and 2 to student demographics, along with 4-class categorical labels. It spans multiple classes and cohorts, making it a structured and heterogeneous dataset ideal for FL experimentation.

**Table 1:** Data set statistics.

Category	Details					Total
	Grade	Class 1	Class 2	Class 3	Class 4	
Grade enrollment	2021	41	40	43	42	166
	2022	39	42	41	41	163
	2023	39	40	43	42	164
Gender distribution	Male	252				
	Female	241				
Data set overview	Total Students	493				
	Total Records	2,452				
	Feature Dimensions	17				
	Labels	Fail, Passed, Good, Excellent				

Participation in this study was voluntary, and all students were fully informed of the research objectives before data collection. The study was approved by the Ethics Committee of the School of Education, Baoji University of Arts and Sciences (Approval No.: BJWLXY-2024-023). All data were anonymized immediately after collection by removing identifiable information such as names and class designations, and each record was assigned a unique code to protect student privacy.

The dataset, sourced from a Python programming course at a single institution and focused on students from a single academic major of Electronic Engineering, may not fully capture the diversity of broader educational contexts. Although the EADP-FedAvg method has been thoroughly validated in this specific setting (see Section 5.2), future research will integrate datasets from multiple institutions and diverse disciplines to enhance the generalizability of the proposed approach.

### 3.2 Data preprocessing

To ensure the quality of the Python Programming online test dataset and improve compatibility with the MLP model used in EADP-FedAvg, this study applied four preprocessing steps to the 2,452 valid records: data cleaning, feature extraction, normalization, and data splitting. These steps addressed missing values, outliers, and discrepancies in feature scale.

#### 3.2.1 Data cleaning

Out of the initial 2,465 raw records, 13 invalid entries were removed. These included records missing programming scores, submissions with response times under 5 min, duplicate entries, and records containing abnormal values. Additionally, error logs not related to Python syntax were excluded to maintain data consistency. After cleaning, 2,452 records remained for analysis.

#### 3.2.2 Feature extraction

Seventeen features were extracted from the cleaned dataset. These included 5 score-related features (total score, multiple choice, fill-in-the-blank, true/false, and programming), 10 behavior-related features (such as submission count and response time), and 2 demographic features (gender and age). Each feature vector was defined at the individual test attempt level, without temporal aggregation, to align with the tabular input requirements of the MLP model.

#### 3.2.3 Data normalization

Due to the varying scales of different features, z-score normalization was applied to standardize the data:


(1) $$z=\frac{x-\mu }{\sigma }\;$$

In Equation 1, x is the original value, μ is the mean, and σ is the standard deviation. After normalization, the transformed features have a mean of 0 and a standard deviation of 1, which improves model training stability. Gender and categorical label data were excluded from this step, as normalization was unnecessary.

#### 3.2.4 Data splitting

By anonymizing student IDs and test identifiers, the final dataset of 2,452 records was constructed by merging score, behavior, and demographic features. The dataset was split into a training set and a test set at an 8:2 ratio, resulting in 1,961 training samples and 491 test samples, following standard practices in educational data mining. The training data was distributed across 10 clients, with each client receiving approximately 196 records. To simplify training, independent and identically distributed (IID) sampling was used. The test set was reserved for global evaluation.

### 3.3 EADP-FedAvg

#### 3.3.1 Theoretical foundation

FL enables multiple clients to collaboratively train a global model without sharing their raw data. By relying on local updates and model parameter aggregation, FL inherently supports privacy preservation and is well-suited for distributed educational data scenarios like the one in this study.

However, despite its decentralized structure, FL still presents potential privacy vulnerabilities. Prior research has demonstrated that adversaries can exploit uploaded model parameters or gradients to infer characteristics of the original training data, and in some cases, even reconstruct parts of the raw samples. These attacks are known as gradient inversion attacks or model reconstruction attacks. The fundamental cause lies in the fact that model parameters are directly influenced by training data; thus, they carry imprints of individual samples, especially when client datasets are small or the model architecture is complex. This increases the risk that updates may leak identifiable personal features.

As a result, the structural design of FL alone is insufficient to guarantee complete privacy. To strengthen the privacy protection mechanism, techniques such as DP can be employed. DP introduces randomness to model updates in each training round, statistically masking the influence of any single data point and reducing the identifiability embedded in model parameters.

Differential Privacy achieves this by injecting noise into model outputs or parameters, ensuring that the probability distributions of algorithm outputs over neighboring datasets remain statistically similar. The formal DP definition is shown in Equation 2:


(2) $$P_{r}[S(D)\in T]\leq e^{\varepsilon }\cdot P_{r}[S(D\prime )\in T]+\delta$$

Here, *ε* is the privacy budget that quantifies the strength of the privacy guarantee, and *δ* is the failure probability. *S(D)* and *S(D′)* denote the algorithm’s output distributions over two neighboring datasets, *D* and *D′*. Traditional DP-FedAvg methods apply fixed Gaussian noise with the following standard deviation (Equation 3):


(3) $$\sigma =\frac{\mathrm{\Delta f}\cdot \sqrt{2\ln(\frac{1.25}{\delta })}}{\varepsilon }\;$$

Where *Δf* represents the sensitivity of the function. However, using fixed noise often compromises model performance, especially in small-scale or heterogeneous educational data settings. To address this limitation, EADP-FedAvg introduces an entropy-adaptive mechanism. It dynamically adjusts the noise intensity based on the average information entropy of the model’s predictions on a test dataset. The adjustment function is shown in Equation 4:


(4) $$\sigma =\frac{\mathrm{\Delta f}\cdot \sqrt{2\ln(\frac{1.25}{\delta })}}{\varepsilon }\cdot (\frac{1}{\mathrm{avg}\_\text{entropy}})\;$$

Where avg_entropy represents the average entropy of predicted probability distributions, calculated as Equation 5:


(5) $$\mathrm{avg}\_\text{entropy}=-\sum p_{i}\log(p_{i})\;$$

The fixed privacy budget (ε = 0.1, δ = 1e-6) ensures consistent privacy protection across all clients but may not fully accommodate varying privacy needs based on data sensitivity or client heterogeneity. A personalized privacy budget, dynamically adjusting ε per client or training phase, could further optimize the privacy-performance trade-off, as explored in future work.

When entropy is high, noise is reduced to improve performance; when entropy is low, noise is increased to enhance privacy protection.

#### 3.3.2 Algorithm design

The EADP-FedAvg algorithm proposed in this study is based on the classic FedAvg framework, with the addition of entropy-adaptive Gaussian noise in the global aggregation phase to enhance differential privacy protection. The core procedure is as follows: first, the server randomly initializes the global model parameters and distributes them to 10 clients, each containing approximately 196 local training samples. Next, each client trains a local MLP model using its data and predefined hyperparameters, completing one local update. Third, each client computes the information entropy based on the predicted probability distribution of its local model on the test data. The server then collects all clients’ entropy results and calculates the global avg_entropy. In the fourth step, the server aggregates the uploaded model parameters and adds Gaussian noise adaptively based on the current avg_entropy value to achieve differential privacy protection. This process is repeated for a total of 200 communication rounds.

The EADP-FedAvg algorithm in this study is shown in Table 2:

**Table 2:** EADP-FedAvg algorithm.

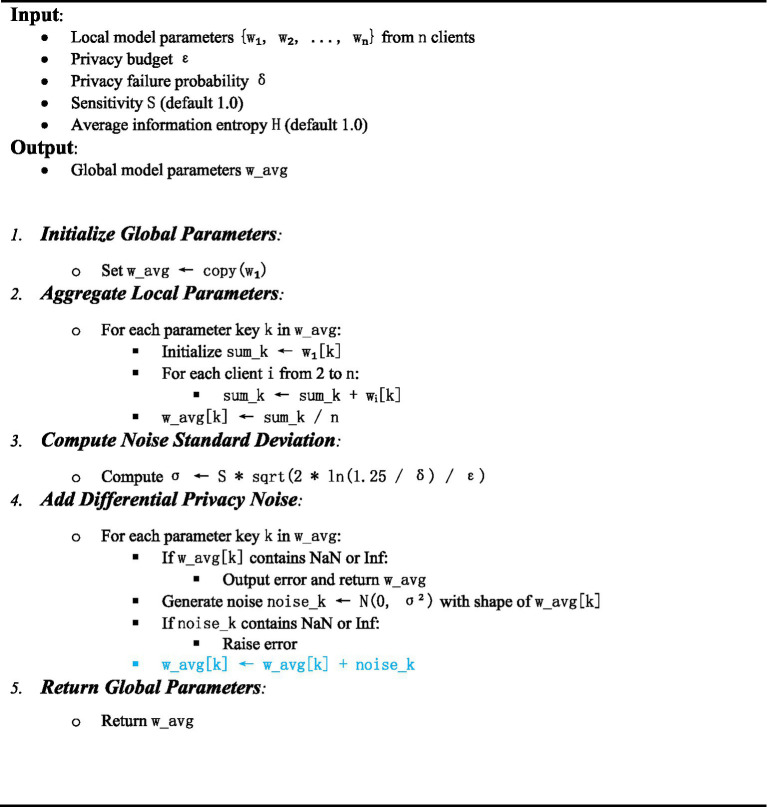

## 4 Experimental design

### 4.1 Experimental setup

Experiments were conducted on a high-performance computing platform simulating a FL environment with multiple collaborating clients. To improve training efficiency and model convergence speed, the hardware setup included a multi-core CPU and sufficient RAM to ensure smooth parallel computation. On the software side, the PyTorch framework was used, along with techniques such as data loading acceleration, gradient clipping, and the Adam optimizer to enhance training stability and performance. The system configuration is detailed in Table 3:

**Table 3:** Experimental environment settings.

	Environment	System parameters
Hardware	CPU	Intel Core i7-9850H, @4.6 GHz, 6核
	RAM	64GB
	GPU	NVIDIA GeForce RTX 2080(8GB GDDR6)
Software	Operating system	Windows 10, 64bit
	Virtualization	VMware Workstation 16
	Python	Python 3.7
	ML Framework	Pytorch 1.9.1

The hardware setup includes a 6-core, 12-thread Intel Core i7-9850H processor with a maximum turbo frequency of 4.6 GHz, 64 GB of RAM, and a dedicated NVIDIA GeForce RTX 2080 GPU. This configuration effectively supports high-performance parallel training of multiple clients and accelerates model convergence. The software environment runs on 64-bit Windows 10, with the EADP-FedAvg algorithm and MLP model implemented using Python 3.7 and PyTorch 1.9.1. VMware Workstation 16 is used to simulate isolated client environments for distributed FL. The Python environment is equipped with commonly used libraries such as NumPy, SciPy, and Scikit-learn for feature engineering, model evaluation, and statistical analysis. PyTorch offers efficient tensor operations and automatic differentiation to speed up MLP model training and optimization.

The EADP-FedAvg experiment uses 10 clients, each performing local training with an MLP model. The model architecture includes 17 input features, two hidden layers with 128 and 64 neurons respectively, and a final output layer producing probabilities for four classes which is suitable for tabular classification tasks. Training parameters were carefully tuned to balance convergence speed and predictive performance. DP is ensured via the Gaussian mechanism, with noise standard deviation dynamically computed using the entropy-adaptive formula detailed in Section 3.3.1.

The use of 10 clients is intended to strike a balance between computational efficiency and representativeness of data distribution, ensuring each client receives a sufficient number of training samples. This supports stable local training, mitigates biases due to data imbalance, and enhances both model robustness and experimental reproducibility.

The full experimental parameter settings are provided in Table 4.

**Table 4:** Experimental parameter settings.

	Parameter	Value	Description
FL parameters	Number of clients	10	Simulates distributed training data across 12 classes
	Global epochs	200	Total number of training epochs
	Local iterations	5	Number of local training rounds before each global aggregation
	Batch size	10	Number of samples per batch in local training
	Optimizer	SGD	–
	Learning rate	0.01	–
	Momentum	0.5	Speeds up convergence and prevents oscillation
	Loss function	Cross-entropy loss	Suitable for 4-class classification tasks
	Entropy computation	Client-side prediction entropy → Server average	Each client calculates entropy based on predicted probabilities on the test data, then uploads to the server for aggregation
Differential privacy parameters	Privacy budget ε	0.1	Strength parameter for differential privacy
	Tolerance probability δ	1.00E-06	Acceptable probability of privacy breach
	Sensitivity Δf	1	Sensitivity based on standardized features
Noise mechanism parameters	Noise std. deviation σ	Dynamically adjusted using entropy-adaptive formula	See Section 3.3.1 for details

To comprehensively evaluate the performance of EADP-FedAvg, two baseline models are set up for comparison. The first is the conventional FL model, which uses the classical FedAvg algorithm. It shares the same MLP architecture and training parameters but does not apply any differential privacy mechanisms. This helps assess the performance of FL without entropy-adaptive enhancements. The second baseline is a centralized learning model, where the MLP is trained directly on the complete dataset of 2,452 records without any privacy protection. This serves as the theoretical upper bound in terms of model performance.

The experiment compares EADP-FedAvg with these two baselines across three evaluation metrics on the test set: accuracy, macro-average score, and average information entropy. The primary goal is to demonstrate that EADP-FedAvg can achieve strong model performance while providing privacy protection under a privacy budget of *ε* = 0.1. To ensure result stability and reliability, each experiment is repeated 20 times, and the average results are reported to mitigate the effects of randomness.

### 4.2 Model and parameters

#### 4.2.1 Model architecture

To accommodate the tabular data structure of the Python Programming course, the EADP-FedAvg method employs a Multi-Layer Perceptron (MLP) model. MLP is a type of feedforward neural network known for its simplicity and efficiency, particularly well-suited for structured data in multi-class classification tasks (Witten et al., 2016). The choice of MLP is motivated by the dataset’s static tabular format, with 17 features (5 score features, 10 behavioral features, and 2 demographic features) that do not explicitly include time-series information, enabling efficient modeling with low computational complexity. However, behavioral features like login frequency may contain latent temporal patterns (e.g., sequential login activities over the course duration), which MLP cannot fully capture, as discussed in Section 6. The model architecture used in this study is as follows:

1. Input Layer: 17 features including 5 score features, 10 behavioral features, and 2 demographic features.
2. Hidden Layer 1: 128 neurons with ReLU activation to capture non-linear relationships among features.
3. Hidden Layer 2: 64 neurons with ReLU activation to further extract high-level features.
4. Output Layer: 4 neurons corresponding to the 4 classification labels, outputting class probabilities.

The forward propagation process of the MLP consists of three sequentially connected linear layers. Between the first two layers, ReLU activation functions are introduced to enhance the model’s non-linear expression capability. First, the input data passes through the first linear transformation, followed by ReLU activation to produce the output of Hidden Layer 1. This output then proceeds through the second linear transformation and another ReLU activation, resulting in the output of Hidden Layer 2. Finally, this output is fed into the third linear layer to produce the model’s final predictions.

The MLP architecture is relatively simple and has low computational complexity, making it well-suited for small-scale educational data as in this study. It processes 17-dimensional vectors directly, and the total number of trainable parameters in this MLP model is 10,820.

#### 4.2.2 Detailed training procedure

The training process of EADP-FedAvg is based on the Federated Averaging algorithm, with the addition of entropy-adaptive Gaussian noise during the global aggregation phase. To isolate the effect of the entropy-adaptive mechanism and ensure experimental controllability, we assume independently and IID data across the 10 clients, enabling consistent statistical properties. However, in real-world educational settings, client data are often non-IID due to heterogeneous student behaviors such as varying login patterns or learning paces, which may impact global model convergence, as discussed in Section 6.

The steps are as follows:

1.  Global Initialization. The central server first initializes the MLP model parameters and randomly distributes the same initial weights to 10 clients.
2.  Local Training. Each client trains its MLP model using local data, optimizing with the cross-entropy loss function (Equation 6):

(6) $$L=-\frac{1}{n}\sum [y_{i}\log(\hat{y_{i}})+(1-y_{i})\log(1-\hat{y_{i}})]\;$$

Which $y_{i}$ represents the true label, $\hat{y_{i}}$ represents the predicted probability, and n is the number of samples.

3.  Entropy Computation. Each client computes the information entropy based on the predicted probability distribution of its local model on the test data. These entropy values are sent to the server and averaged to generate the global average entropy, as described in Section 3.3.1.
4.  Global Aggregation. The server receives the locally trained model parameters from the 10 clients and performs a weighted average to update the global model. To enhance privacy protection, entropy-adaptive Gaussian noise is added to the aggregated parameters in accordance with differential privacy constraints (Equation 7). The noise standard deviation and addition mechanism are detailed in Section 3.3.1. The noise is added to each layer’s aggregated weights:

(7) $$w_{\mathrm{avg}}[k]=\mathrm{dp}\_\mathrm{add}\_\text{noise}(w_{\mathrm{avg}}[k],\sigma )$$

Which $w_{\mathrm{avg}}[k]$ represents the aggregated weights of the k−th layer, and the dp_add_noise function adds Gaussian noise to those parameters.

5.  Model Broadcasting. The updated global parameters are broadcast back to each client to commence the next training round.
6.  Epochs Termination. The global training proceeds for 200 epochs until completion.

#### 4.2.3 Parameter settings

Key parameters of the MLP and EADP-FedAvg were tuned to ensure convergence and privacy protection, as summarized in Table 5:

**Table 5:** Overview of EADP-FedAvg and MLP parameter configuration.

Category	Parameter name	Value or description
Model parameters	Input dimension	17
	Output dimension	4
	Activation function	ReLU
	Hidden layer structure	
	├─ Layer 1	Linear(17 → 128), ReLU
	├─ Layer 2	Linear(128 → 64), ReLU
	└─ Output layer	Linear(64 → 4 或 2), No activation
Training parameters	Number of clients	10
	Global communication epochs	200
	Local iterations per Client	5
	Batch size	10
	Learning rate	0.01
	Momentum	0.5
	Loss function	Cross Entropy
Privacy parameters	Privacy budget	ε = 0.1, δ = 1e^−6^
	Sensitivity	∆*f* = 1.0
	Noise	Gaussian Mechanism

## 5 Experimental results and analysis

### 5.1 Comparative analysis of the training

Performance evaluation employs the following metrics:

1.  Accuracy. The proportion of correctly predicted samples, it is calculated as shown in Equation 8:

(8) $$\text{Accuracy}=\frac{\sum_{i=1}^{k}{\mathrm{TP}}_{i}}{\sum_{i=1}^{k}({\mathrm{TP}}_{i}+{\mathrm{TN}}_{i}+{\mathrm{FP}}_{i}+{\mathrm{FN}}_{i})}\;$$

2.  Training Loss. Cross-entropy loss, measuring model convergence (formula in Section 4.2.2).
3.  Average Information Entropy. Quantifies the uncertainty of the predictions and guides the noise adjustment in EADP-FedAvg (formula in Section 3.3.1).

Each of the three models was trained on the training set over 20 independent random trials, with each trial running for 200 epochs. The training data presented here represent the average results from these 20 trials.

**Figure 1:**
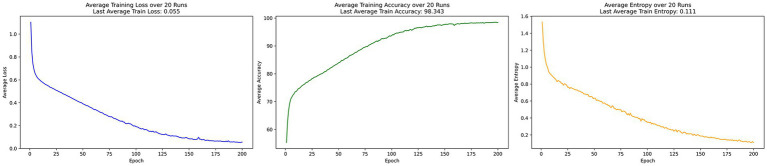
Centralized learning training process.

In the centralized learning training process shown in Figure 1, the model’s loss converges to 0.055, indicating excellent optimization. Accuracy stabilizes around the 150th epoch, reaching 98%. After 200 epochs, the average information entropy remains low at 0.111, demonstrating the model’s strong grasp of the data distribution and efficient fitting.

**Figure 2:**
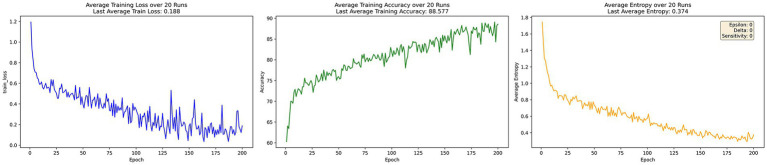
DP-FedAvg training process.

Figure 2 illustrates the training performance of DP-FedAvg under privacy protection mechanisms. The model’s loss converges at 0.188, noticeably higher than centralized learning. Accuracy reaches 88.577% after 200 epochs. However, due to the additional noise introduced by differential privacy, the average information entropy increases to 0.374, indicating increased uncertainty in predictions despite maintaining reasonable accuracy.

**Figure 3:**
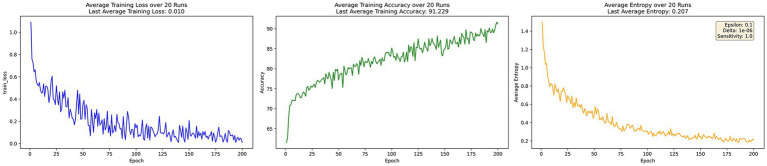
EADP-FedAvg training process.

Figure 3 presents the training process of EADP-FedAvg, which incorporates entropy-adaptive noise adjustment. Under this improved strategy, the model loss converges faster to just 0.01. Accuracy improves to 91.229% at 200 epochs, showing a clear enhancement compared to DP-FedAvg. Meanwhile, average information entropy drops to 0.207, reflecting a better balance between privacy protection and model performance achieved by the entropy-adaptive mechanism.

Overall, the experimental results demonstrate that EADP-FedAvg outperforms DP-FedAvg with superior convergence, achieving lower training loss and higher accuracy after 200 epochs. This improvement stems from the entropy-adaptive noise mechanism, which dynamically reduces noise interference when prediction uncertainty is high, thereby preserving more useful information. Although centralized learning attains the highest accuracy and lowest entropy with minimal loss, it lacks any data privacy protection. In contrast, EADP-FedAvg effectively balances privacy protection and convergence efficiency while maintaining strong model performance.

### 5.2 Comparative analysis in the testing

To further compare the generalization performance of the three models in multi-class classification tasks, Tables 6–8 present the confusion matrix results on the test set for centralized learning, DP-FedAvg, and EADP-FedAvg, respectively. The four predicted labels—Fail, Passed, Good, and Excellent—correspond to students’ performance levels in the course. Below each confusion matrix, the Precision for each class is provided to evaluate the model’s local prediction accuracy.

**Table 6:** Confusion matrix for centralized learning.

		**Predicted**			
		**Fail**	**Passed**	**Good**	**Excellent**
Actual	Fail	69	2	1	0
	Passed	1	144	0	3
	Good	2	3	173	2
	Excellent	0	0	1	90
	Total	72	149	175	95
	Precision	0.958	0.966	0.989	0.947

**Table 7:** Confusion matrix for DP-FedAvg.

		**Predicted**			
		**Fail**	**Passed**	**Good**	**Excellent**
Actual	Fail	60	7	4	1
	Passed	5	131	7	5
	Good	3	8	158	11
	Excellent	1	1	3	86
	Total	69	147	172	103
	Precision	0.870	0.891	0.919	0.835

**Table 8:** Confusion Matrix for EADP-FedAvg.

		**Predicted**			
		**Fail**	**Passed**	**Good**	**Excellent**
Actual	Fail	68	3	1	0
	Passed	4	135	6	3
	Good	2	5	164	9
	Excellent	0	1	2	88
	Total	74	144	173	100
	Precision	0.919	0.938	0.948	0.880

From the results, the centralized learning model demonstrates the highest accuracy across all categories. In particular, the *Good* and *Passed* categories achieve Precisions of 0.989 and 0.966, respectively, reflecting superior overall predictive performance with minimal misclassification.

In contrast, the performance of the DP-FedAvg model drops notably after introducing fixed-intensity noise (*ε* = 0.1). Precision for the *Fail* class falls to 0.87, and for the *Excellent* class to 0.835, suggesting reduced stability in predictions under differential privacy constraints. The EADP-FedAvg model, on the other hand, incorporates an entropy-adaptive noise mechanism. By dynamically adjusting noise levels while maintaining the same privacy budget, it improves predictive accuracy. In the *Good* and *Passed* classes, Precisions reach 0.948 and 0.938 respectively, which is significantly better than DP-FedAvg and approaching the level of centralized learning.

The final summary of experimental results is as follows:

Table 9 provides an overview of the overall performance of the three models. The centralized model ranks highest in accuracy (0.969), macro average score (0.965), and lowest average entropy (0.111), but it lacks any privacy-preserving capability.

**Table 9:** Summary of performance comparison.

Model	Accuracy	Macro average score	Average entropy
Centralized machine learning	0.969	0.965	0.111
DP-FedAvg	0.886	0.879	0.374
EADP-FedAvg	0.927	0.921	0.207

The DP-FedAvg model shows decreased accuracy (0.886) and increased average entropy (0.374), indicating higher uncertainty in predictions under privacy constraints.

EADP-FedAvg demonstrates notable improvements, with accuracy rising to 0.927 and macro average score to 0.921, while average entropy drops to 0.207—validating the effectiveness of the entropy-adaptive mechanism in enhancing model robustness and performance.

Moreover, EADP-FedAvg shows a marked improvement in recognizing high-performing students (*Excellent* category), with recall increasing by approximately 4% compared to DP-FedAvg. This is particularly important in evaluation systems, such as programming courses, where high-end performance matters significantly.

By dynamically adjusting the noise scale based on average entropy, the model achieves an effective trade-off between privacy preservation and predictive performance. This design demonstrates strong stability in heterogeneous data environments—such as those combining academic scores and behavioral features—and offers promising potential for generalization and privacy adaptability.

## 6 Conclusion

This study proposed an EADP-FedAvg, designed to predict student performance in engineering education scenarios. The dataset was collected from the School of Physics and Optoelectronic Technology at Baoji University of Arts and Sciences, covering students majoring in Electronic Engineering from cohorts 2021 to 2023. It includes 2,452 records from online assessments in the Python Programming course. Under a strict privacy budget (*ε* = 0.1, *δ* = 1e-6), the proposed model successfully protected individual student data while achieving high predictive performance. On the test set, EADP-FedAvg attained an accuracy of 92.7%, significantly outperforming the conventional DP-FedAvg (88.6%) and approaching the performance upper bound of centralized machine learning (96.9%), demonstrating both adaptability and practical utility.

The core advantage of EADP-FedAvg lies in its integration of FL’s distributed architecture with an entropy-adaptive differential privacy mechanism. The model dynamically reduces the intensity of added noise during high-entropy phases, thereby minimizing disruption to training, while increasing noise in low-entropy phases to reinforce privacy protection. This effectively mitigates the performance degradation commonly caused by static-noise mechanisms. As shown in Section 5.1, after 200 training rounds, the final loss of the EADP-FedAvg model converged to 0.01, and its accuracy reached 91.229%, outperforming DP-FedAvg’s final loss of 0.188 and accuracy of 88.577%. Moreover, the model’s average information entropy significantly decreased to 0.207—substantially lower than DP-FedAvg’s 0.374—indicating greater predictive certainty and model stability, thereby confirming the practical effectiveness of EADP-FedAvg’s theoretical foundation.

Despite the promising results, the study has several limitations. First, the dataset, sourced from a Python programming course at a single institution and focused on students from a single major, may not fully capture the diversity of broader educational contexts. Although the dataset includes 2,452 records and the EADP-FedAvg method has been rigorously validated in this specific setting, achieving a test accuracy of 92.7%, its generalizability to other institutions or disciplines remains to be explored. Second, the use of a fixed privacy budget (*ε* = 0.1, *δ* = 1e-6) ensures consistent privacy protection across all clients but may not fully accommodate varying privacy needs based on data sensitivity, client heterogeneity, or task requirements. This uniform approach, while effective in achieving 92.7% test accuracy, could introduce excessive noise for less sensitive data or insufficient protection for highly sensitive data, potentially impacting model performance in diverse educational scenarios. Third, the model is based on an MLP architecture, which, while effective for the static tabular data used in this study, lacks the capability to model temporal dependencies inherent in students’ learning behaviors, such as sequential patterns in login activities or performance trends over the course duration. Moreover, the study compares only three models, including centralized machine learning, DP-FedAvg, and EADP-FedAvg, all using the MLP architecture, as shown in Table 9. This limits the exploration of diverse architectures and federated learning algorithms, potentially restricting the evaluation of EADP-FedAvg’s robustness across varied settings. Finally, for experimental simplicity, the study assumes independently and IID data across the 10 clients for training and testing, as detailed in Section 4.2.2. This assumption, while enabling controlled evaluation of EADP-FedAvg’s entropy-adaptive mechanism, does not address the more realistic non-IID distribution prevalent in federated learning, where client data may exhibit heterogeneous distributions. Such heterogeneity could degrade global model convergence or introduce biased predictions in real-world educational settings.

To address these limitations, future work can explore several directions for improvement and extension. First, to enhance the generalizability of EADP-FedAvg, we plan to validate the model across datasets from multiple institutions and diverse academic disciplines, such as Computer Science, Mechanical Engineering, and Mathematics, as well as different course types like Java Programming or Data Science, to ensure robustness across varied educational contexts, as detailed in Supplementary Table S1. Second, to address varying privacy needs, we plan to develop a dynamic privacy budget control mechanism, adjusting *ε* based on factors such as data sensitivity, client-specific entropy levels, or training phases, to optimize the privacy-performance trade-off across heterogeneous educational datasets, as outlined in Supplementary Table S4. Third, to capture temporal dependencies in student behavior data, we plan to explore alternative model architectures, such as Recurrent Neural Networks (RNNs) or Transformers, to model sequential patterns like login sequences or performance trends over the course duration, and compare these with MLP and centralized models, as detailed in Supplementary Table S3. Fourth, to address the non-IID nature of real-world educational datasets, we plan to develop personalized federated learning algorithms, such as client clustering based on behavioral similarity or local model fine-tuning with meta-learning techniques, such as Model-Agnostic Meta-Learning (MAML). These approaches will mitigate the impact of heterogeneous student behaviors on global model convergence. Finally, to enhance deployment efficiency, we plan to explore techniques such as asynchronous updates, model compression, and gradient sparsification. To further enhance transparency while adhering to privacy constraints, we plan to explore sharing aggregated or synthetic datasets compliant with China’s Personal Information Protection Law (PIPL) and ethical guidelines, as detailed in Supplementary Table S5. All detailed enhancement plans are provided in the Supplementary material document.

The proposed EADP-FedAvg approach achieves a desirable balance between accuracy and privacy preservation, offering a practical path for privacy-aware modeling and intelligent learning analytics in engineering education. Future research will continue to enhance the model’s generalization capabilities, improve temporal modeling, and expand support for personalized privacy mechanisms, advancing the application of FL in the domain of intelligent education systems.

## Data Availability

The datasets presented in this article are not readily available because the datasets (2,452 records from a Python programming course at Baoji University of Arts and Sciences) contain sensitive student information and are protected under China’s Personal Information Protection Law (PIPL) and institutional ethical guidelines (Approval No.: BJWLXY-2024-023), precluding public availability. Researchers may request access to the anonymized dataset by contacting the Ethics Committee of the School of Education, Baoji University of Arts and Sciences or the correspondence author, subject to approval and data use agreements. Requests to access the datasets should be directed to webmaster@bjwlxy.edu.cn or Shanwei Chen, chenshanwei@bjwlxy.edu.cn.

## References

[ref1] Afrose S. Hashem T. Ali M. E. (2021). “Frequent itemsets mining with a guaranteed local differential privacy in small datasets” in Proceedings of the 33rd international conference on scientific and statistical database management.

[ref2] Bonawitz K. Kairouz P. McMahan B. Ramage D. (2021). Federated learning and privacy: building privacy-preserving systems for machine learning and data science on decentralized data. Queue 19, 87–114. doi: 10.1145/3494834.3500240

[ref3] Chicaiza J. Cabrera-Loayza M. C. Elizalde R. Piedra N. (2020). “Application of data anonymization in learning analytics” in Proceedings of the 3rd international conference on applications of intelligent systems.

[ref4] Christiansen S. H. Juebei C. Xiangyun D. (2023). Cross-institutional collaboration in engineering education–a systematic review study. Eur. J. Eng. Educ. 48, 1102–1129. doi: 10.1080/03043797.2023.2228727

[ref5] Elgabli A. Mesbah W. (2024). A novel approach for differential privacy-preserving federated learning. IEEE Open J. Commun. Soc. 1, 1–10. doi: 10.1109/OJCOMS.2024.3521651

[ref6] Khan S. (2024). Secure multi-party computation for privacy preservation in big data analytics. J. Big Data Privacy Manage. 2, 168–179. Available at: https://jbdpm.com/index.php/journal/article/view/42

[ref7] Liu T. (2024). “Research on privacy techniques based on multi-party secure computation” in 2024 3rd international conference on artificial intelligence and autonomous robot systems.

[ref8] Marshall R. Pardo A. Smith D. Watson T. (2022). Implementing next generation privacy and ethics research in education technology. Br. J. Educ. Technol. 53, 737–755. doi: 10.1111/bjet.13224

[ref9] McMahan H. B. Yu F. X. Richtarik P. Suresh A. T. Bacon D. (2016). “Federated learning: Strategies for improving communication efficiency” in Proceedings of the 29th conference on neural information processing systems, Barcelona, Spain.

[ref10] Miller J. Chattopadhyay A. (2024). “Integrating differential privacy in modern database curriculum” in 2024 IEEE integrated STEM education conference.

[ref11] Mora A. Bujari A. Bellavista P. (2024). Enhancing generalization in federated learning with heterogeneous data: a comparative literature review. Futur. Gener. Comput. Syst. 1, 1–10. doi: 10.1016/j.future.2024.03.027

[ref12] Moshawrab M. Adda M. Bouzouane A. Ibrahim H. Raad A. (2023). Reviewing federated learning aggregation algorithms; strategies, contributions, limitations and future perspectives. Electronics 12, 1–35. doi: 10.3390/electronics12102287, PMID: 40789738

[ref13] Pakina A. K. Pujari M. (2024). Differential privacy at the edge: a federated learning framework for GDPR-compliant TinyML deployments. IOSR J. Comput. Engin. 26, 52–64. doi: 10.9790/0661-2602045264

[ref14] Salas J. Domingo-Ferrer J. (2018). Some basics on privacy techniques, anonymization and their big data challenges. Math. Comput. Sci. 12, 263–274. doi: 10.1007/s11786-018-0344-6

[ref15] Sattler F. Wiedemann S. Müller K.-R. Samek W. (2019). Robust and communication-efficient federated learning from non-iiD data. IEEE Transact. Neural Networks Learn. Syst. 31, 3400–3413. doi: 10.1109/TNNLS.2019.2944481, PMID: 31689214

[ref16] Shou Z. Xie M. Mo J. Zhang H. (2024). Predicting student performance in online learning: a multidimensional time-series data analysis approach. Appl. Sci. 14:2522. doi: 10.3390/app14062522

[ref17] Song J. Jung H Chun S Lee H Kang M Park M. (2023). How to decentralize the internet: a focus on data consolidation and user privacy. Comput. Netw. 234:109911. doi: 10.1016/j.comnet.2023.109911

[ref18] Truex S.., A hybrid approach to privacy-preserving federated learning, in Proceedings of the 12th ACM workshop on artificial intelligence and security, (2019)

[ref001] Witten I. H. Frank E. Hall M. A. Pal C. J. (2016). Data Mining: Practical Machine Learning Tools and Techniques, 4th ed. Cambridge, MA: Morgan Kaufmann., PMID:

[ref19] Yang X. Huang W. Ye M. (2023). Dynamic personalized federated learning with adaptive differential privacy. Adv. Neural Inf. Proces. Syst. 36, 72181–72192. Available at: https://proceedings.neurips.cc/paper_files/paper/2023/file/e4724af0e2a0d52ce5a0a4e084b87f59-Paper-Conference.pdf

[ref20] Zhan C. Joksimović S. Ladjal D. Rakotoarivelo T. Marshall R. Pardo A. (2024). Preserving both privacy and utility in learning analytics. IEEE Trans. Learn. Technol. 17, 1615–1627. doi: 10.1109/TLT.2024.3393766

[ref21] Zhang J. Cheng X. Yang L. Hu J. Liu X. Chen K. (2024). Sok: fully homomorphic encryption accelerators. ACM Comput. Surv. 56, 1–32. doi: 10.1145/3676955

