# Supplementary material for: Entropy-adaptive differential privacy federated learning for student performance prediction and privacy protection: a case study in Python programming
Source: Front Artif Intell. 2025 Sep 8;8:1653437. doi: 10.3389/frai.2025.1653437 (PMC12450954; doi:10.3389/frai.2025.1653437)
Supplement: Supplementary file 1 [file Table_1.DOCX]

**Supplementary Material**

Supplementary Table S1: Planned Dataset Expansion

To address the limitation of a single-institution dataset (Section 3.1) and enhance generalizability (Section 6), this table outlines plans for dataset expansion across multiple institutions, majors, and courses.

| **Aspect** | **Current Study** | **Future Work** |
| --- | --- | --- |
| **Institution** | Single (Baoji University) | Multiple (e.g., 5+ universities) |
| **Major** | Single (Electronic Engineering) | Multiple (e.g., CS, Mechanical Engineering, Mathematics) |
| **Course** | Python Programming | Diverse (e.g., Java, Data Science) |

Supplementary Table S2: Planned Enhancements for Non-IID Data Handling in EADP-FedAvg

To address the IID assumption limitation (Section 4.2.2) and handle non-IID data in federated learning (Section 6), this table details personalized FL strategies.

| **Aspect** | **Current Study** | **Future Work** |
| --- | --- | --- |
| **Data Distribution** | IID (uniform across 10 clients) | Non-IID (heterogeneous due to diverse student behaviors) |
| **Example** | Same statistical properties (e.g., login frequency distribution) | Varying distributions (e.g., different login patterns or learning paces across students) |
| **Algorithm Strategy** | Standard Federated Averaging | Personalized FL: client clustering (e.g., K-means on behavioral similarity) or local model fine-tuning (e.g., MAML) ;compare with FedProx, SCAFFOLD |
| **Clustering Method** | N/A | Distribution-based clustering (e.g., K-means on feature entropy or login pattern distributions) |
| **Fine-Tuning Approach** | N/A | Meta-learning (e.g., MAML) or local adaptation with weighted personalization layers |
| **Privacy Mechanism** | Gaussian noise (ε = 0.1, δ = 1e-6) | Entropy-adaptive Gaussian noise compatible with personalized FL |
| **Evaluation Metrics** | Classification accuracy (92.7%) | Accuracy per client cluster, convergence rate under non-IID conditions |
| **Implementation Challenge** | None (IID simplifies aggregation) | Handling distribution skew, balancing personalization and global model consistency |

Supplementary Table S3: Planned Enhancements for Temporal Modeling

To overcome the MLP’s limitation in capturing temporal dependencies (Section 4.2.1) and model sequential behaviors (Section 6), this table outlines advanced architectures and preprocessing methods.

| **Aspect** | **Current Study** | **Future Work** |
| --- | --- | --- |
| **Model Architecture** | Multi-Layer Perceptron (MLP) | Recurrent Neural Networks (LSTM, GRU) or Transformers ; compare with MLP (EADP-FedAvg) and centralized ML |
| **Feature Type** | Static tabular (17 features: 5 score, 10 behavioral, 2 demographic) | Time-series (login sequences, assignment submission patterns, performance trends) |
| **Feature Example** | Login frequency (scalar), total logins per student | Sequences of login timestamps or activity counts ([t1, t2, t3] or [count1, count2, count3]) |
| **Preprocessing Method** | None (direct input of 17-dimensional vectors) | Sequence construction (time-based bucketing of login events into fixed intervals, sliding window of performance scores) |
| **Sequence Length** | N/A | Variable,10-50 time steps based on course duration |
| **Time Window** | N/A | Configurable, daily, weekly, or per-assignment intervals |
| **Model Parameters** | 10,820 (MLP: 17→128→64→4, ReLU) | LSTM: 1-2 layers, 128-256 hidden units; Transformer: 2-4 layers, 4-8 attention heads |
| **Privacy Mechanism** | Gaussian noise (ε = 0.1, δ = 1e-6) | Entropy-adaptive Gaussian noise applied to RNN/Transformer gradients |
| **Evaluation Metrics** | Classification accuracy (92.7%, Section 6) | Temporal prediction accuracy (next-event prediction), cross-entropy loss on sequences |
| **Implementation Challenge** | None (static data processing) | Handling variable-length sequences, ensuring privacy-preserving aggregation in FL |

Supplementary Table S4: Planned Enhancements for Privacy Budget To address the fixed privacy budget limitation (Section 3.3.1) and optimize privacy-performance trade-offs (Section 6), this table proposes dynamic privacy budget adjustments.

| **Aspect** | **Current Study** | **Future Work** |
| --- | --- | --- |
| **Privacy Budget** | Fixed (ε = 0.1, δ = 1e-6) | Dynamic (ε adjusted by sensitivity, entropy, or phase) |
| **Adjustment Criteria** | Uniform across clients | Client-specific (e.g., entropy-based, data sensitivity) |
| **Example** | Same ε for all student data | Higher ε for test scores, lower ε for demographic features |

Supplementary Table S5: Dataset Summary for Transparency

To address data sharing restrictions (Section 12) and enhance transparency within PIPL constraints (Section 6), this table summarizes the dataset and access details.

| **Aspect** | **Details** |
| --- | --- |
| **Total Records** | 2,452 |
| **Institutions** | Baoji University of Arts and Sciences |
| **Major** | Electronic Engineering |
| **Course** | Python Programming |
| **Features** | 17 (5 scores, 10 behavioral, 2 demographic) |
| **Labels** | Fail, Passed, Good, Excellent |
| **Access Restrictions** | Restricted due to PIPL and ethical guidelines |
| **Access Request** | Contact chenshanwei@bjwlxy.edu.cn or webmaster@bjwlxy.edu.cn |
